# Supplementary material for: Reactivity mapping of nanoscale defect chemistry under electrochemical reaction conditions
Source: Nat Commun. 2019 Dec 13;10:5702. doi: 10.1038/s41467-019-13692-3 (PMC6910959; doi:10.1038/s41467-019-13692-3)
Supplement: Supplementary file 1 — Supplementary information [file 41467_2019_13692_MOESM1_ESM.pdf]

Supplementary Information:

Reactivity mapping of nanoscale defect chemistry under electrochemical reaction conditions

Pfisterer et al.

Supplementary Figures

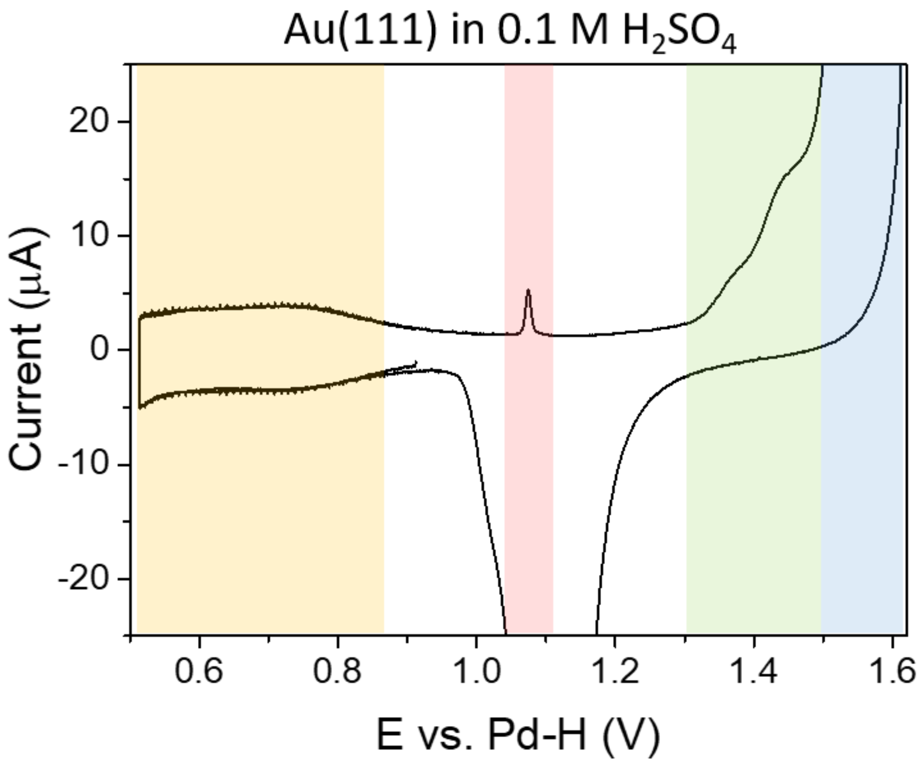

Supplementary Fig. 1 Cyclic voltammetry of Au(111) single crystal. Typical CV of a well-defined Au(111) single crystal in 0.1 M H_2SO_4 recorded in our EC-TERS spectro-electrochemical cell with a scan rate of 50 mV s^{-1}. The sharp peak around 1.08 V vs. Pd-H indicates the order/disorder transition at Au(111) terraces and is characteristic for a well-defined Au(111) single crystal. The green and blue areas highlight the defect and (111) terrace oxidation regions at 1.3-1.48 V vs. Pd-H and 1.48-1.6 V vs. Pd-H, respectively. The cathodic peak at around 1.1 V vs. Pd-H corresponds to the reduction of the AuOx. The highlighted yellow area shows the (bi)sulfate adsorption/desorption region.

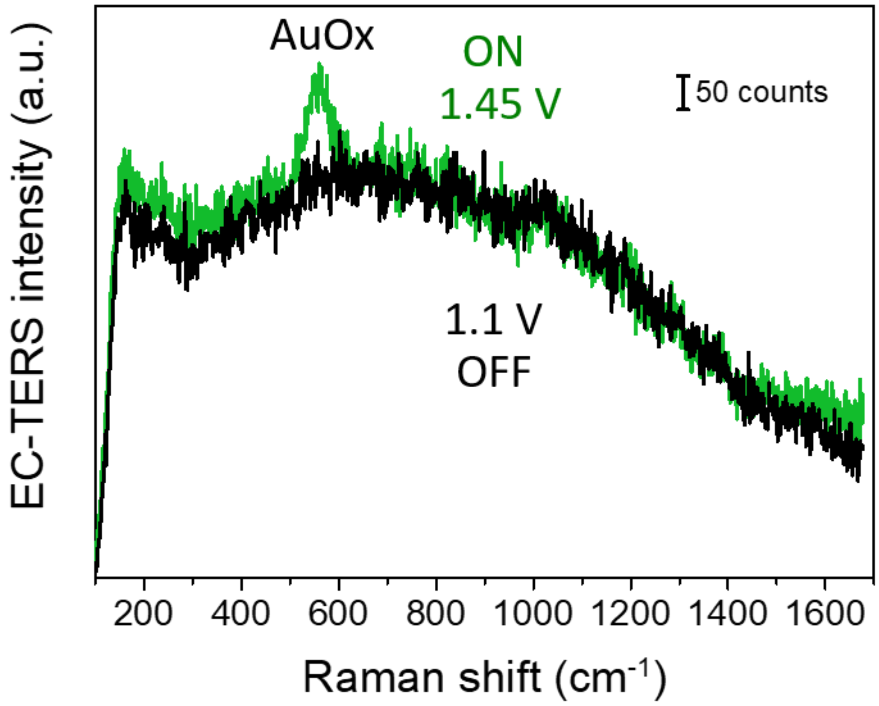

Supplementary Fig. 2 EC-TER spectra for ON and OFF states. EC-TER spectra of Fig. 1c showing the filter cut-off at 156 cm^{-1} and the overall background shape for the ON (1.45 V vs. Pd-H) and OFF (1.1 V vs. Pd-H) states.

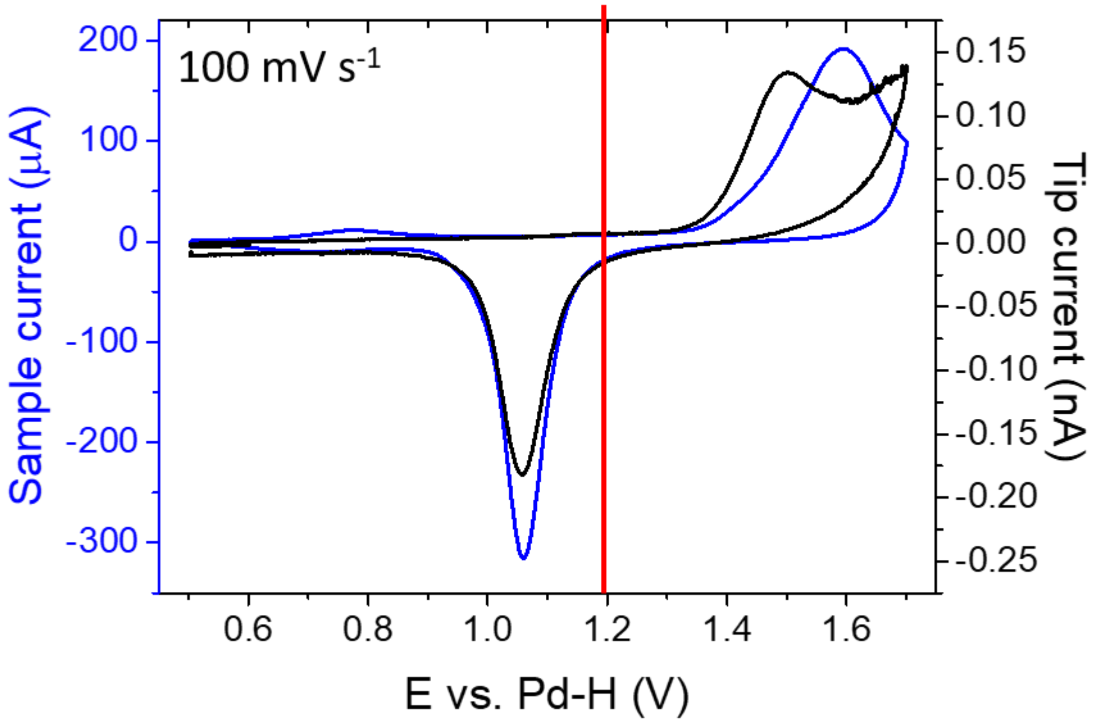

Supplementary Fig. 3 CV of Au(111) and Au-tip. Cyclic voltammograms of the Au(111) sample electrode (blue) and the Au-tip (black) in 0.1 M H_2SO_4. The bias voltage is set to 0 V and the CVs were acquired simultaneously with a scan rate of 100 mV s^{-1}. The red line indicates the maximum Au-tip potential utilized within this work, which ensures that our Au-tip remains AuOx free. Typically the tip potential was fixed at 1.0 V vs. Pd-H.

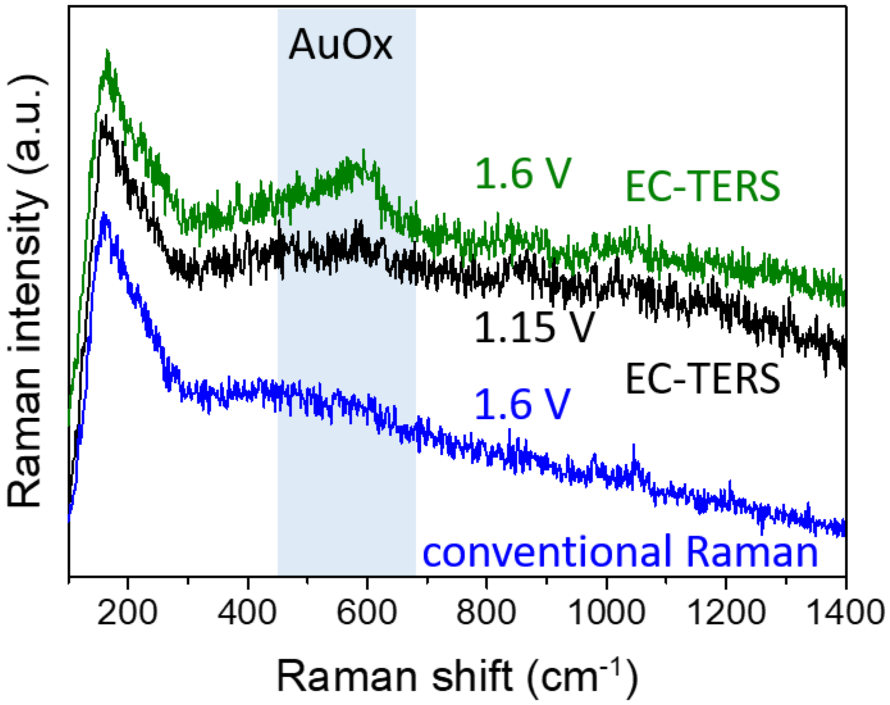

Supplementary Fig. 4 EC-TER spectrum for oxidized terraces. EC-TER spectrum at 1.6 V vs. Pd-H (green) where (111) terrace oxidation is thermodynamically possible, i.e. the complete surface is oxidized. If the tip is retracted from the surface (blue), the conventional far-field Raman spectrum (tip ca. 6 µm retracted from the surface) cannot resolve the AuOx surface species (sample potential still at 1.6 V vs. Pd-H). At 1.15 V vs. Pd-H, when the tip is approached to the surface, no AuOx peak is visible (black). The spectral acquisition time was 5 s. The bias voltage was kept constant at 400 mV, i.e. max. tip potential: 1.2 V vs. Pd-H (at 1.6 V vs. Pd-H applied to sample electrode). The tunneling current setpoint was 1 nA.

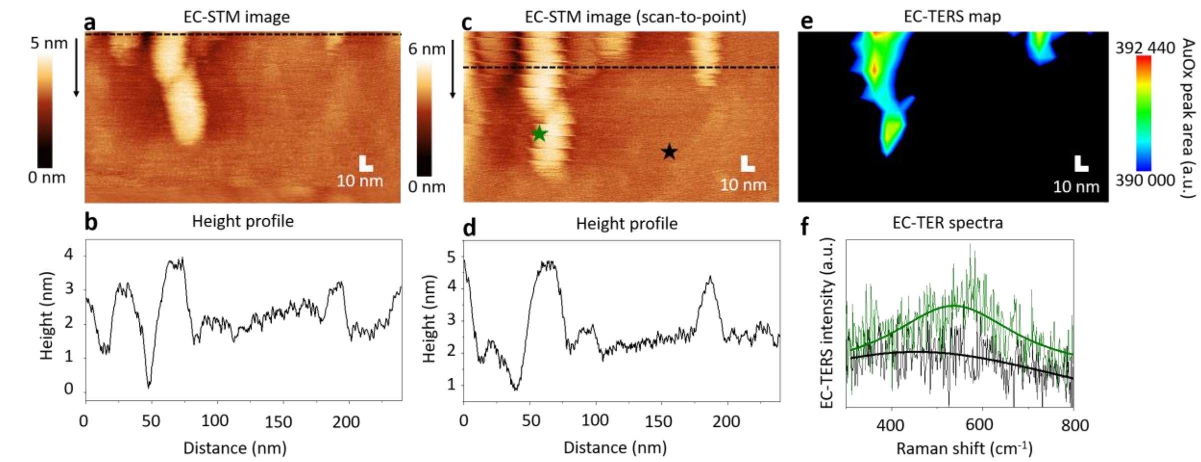

Supplementary Fig. 5 EC-TERS chemical mapping. **a** EC-STM image recorded from top to bottom at 1.45 V vs. Pd-H (tunneling current setpoint: 1 nA, tip potential fixed to 1.0 V vs. Pd-H, 140 nm x 240 nm, scan speed: 1 line s^{-1}) before EC-TERS mapping. **b** Corresponding EC-STM line profile along the dotted black line in **a**. **c** EC-STM image recorded as scan-to-point image while simultaneously acquiring EC-TER spectra at the equidistant “lines” with 9.4 nm distance (mismatch due to thermal drift). Black arrow indicates scan direction. **d** Height profile along the dotted black line in **c**. **e** EC-TERS map indicating the peak area of the 560-580 cm^{-1} AuOx peak as well as changes in EC-TER background (pixel size 9.4 nm, 1 s acquisition time per pixel). The EC-TERS map was created by integrating the AuOx region without background subtraction (cf. Fig. 2 for background-subtracted map). **f** EC-TER spectra recorded during defect oxidation (at 1.45 V vs. Pd-H) at defect site (green) and flat Au region (black), corresponding to the marked green and black stars in **c**. The acquisition time accounted to 3 s. Thick green and black lines represent guides for the eye.

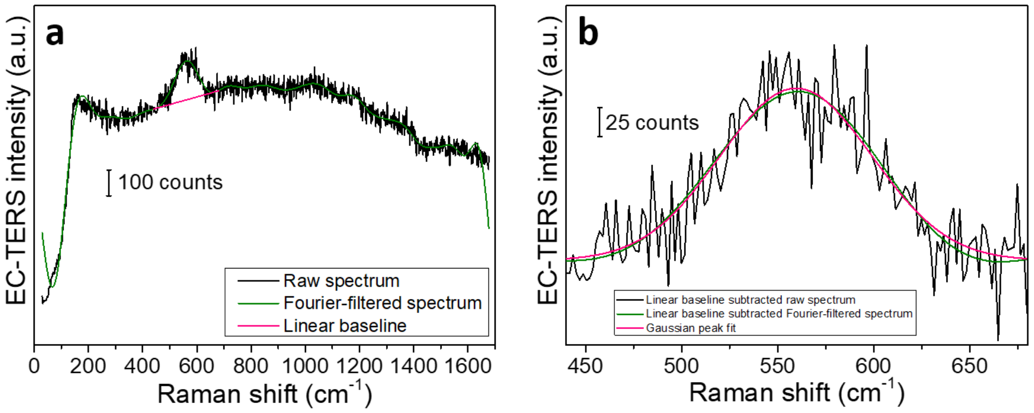

Supplementary Fig. 6 Data treatment for mapping and fitting. **a** Example raw and Fourier-filtered EC-TER spectra (black vs. green) and the linear baseline (pink). **b** Linear baseline subtracted raw and Fourier-filtered EC-TER spectra as well as the single unconstraint Gaussian peak fit.

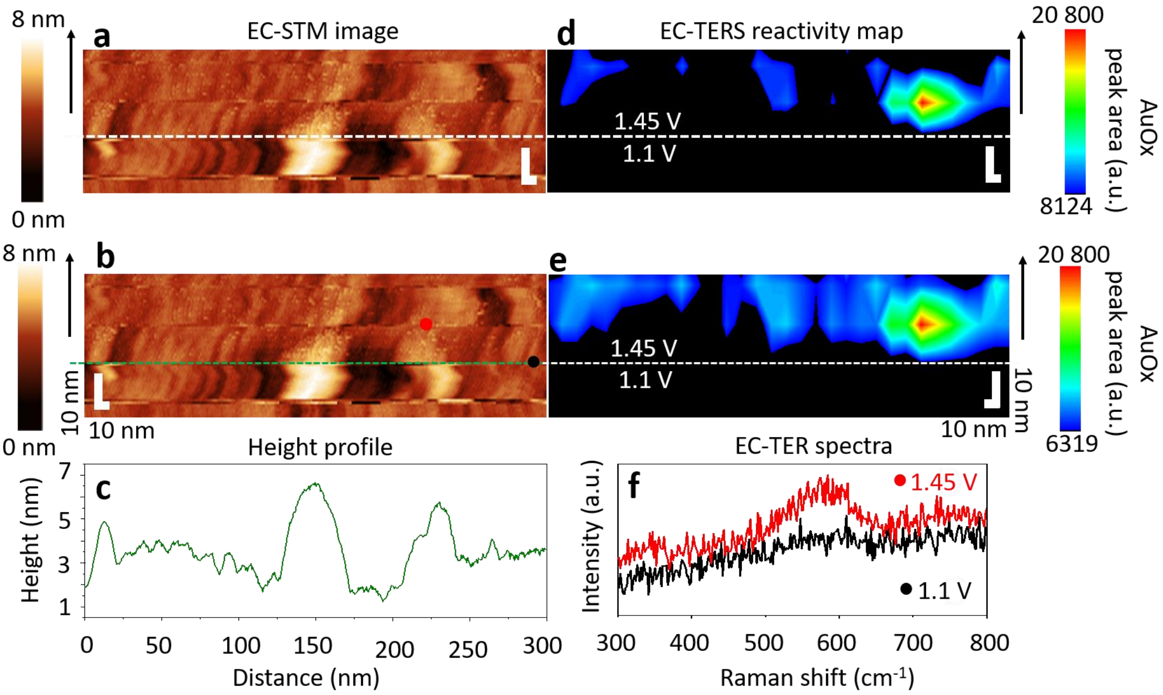

Supplementary Fig. 7 Influence of different scale bar boundaries. **a**,**b** EC-STM image recorded from bottom to top while the sample potential was switched from 1.1 to 1.45 V vs. Pd-H halfway through the image (white and green dotted line). **c** Height-profile along the dotted green line revealing three distinct surface structures. **d**,**e** EC-TERS reactivity map with different lower boundaries emphasizing the most reactive spots on the Au surface. Black arrows indicate scan directions. **f** Example EC-TER spectra at 1.45 V vs. Pd-H (red) and 1.1 V vs. Pd-H (black) at the corresponding red and black marks in **b**.

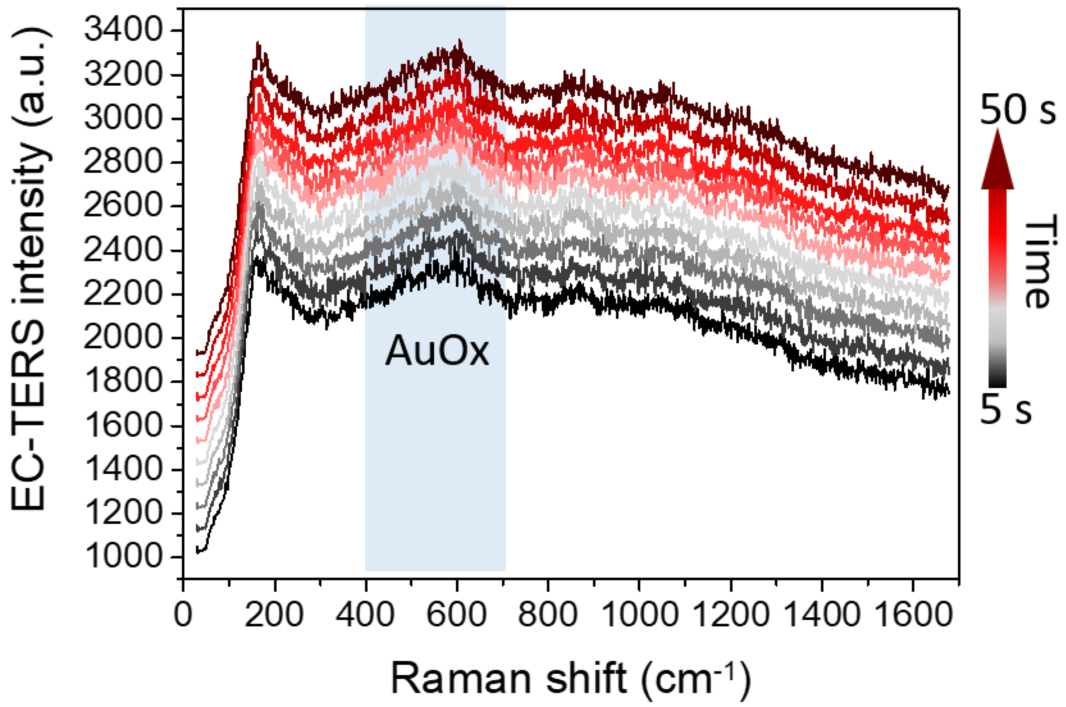

Supplementary Fig. 8 EC-TER spectral time series. EC-TER spectra recorded subsequently at the same spot at 1.6 V vs. Pd-H. All spectra are offset from each other by 200 a.u. for clarity and would otherwise overlap. Each EC-TER spectrum has an acquisition time of 5 s. The tunneling current setpoint was 1 nA and the tip potential was at 1.2 V vs. Pd-H, i.e. 400 mV bias voltage. The broad AuOx region around 560 to 580 cm^{-1} is highlighted in light blue. The colored arrow (black to dark red) represents the spectral time evolution ($\Delta t = 45$ s).

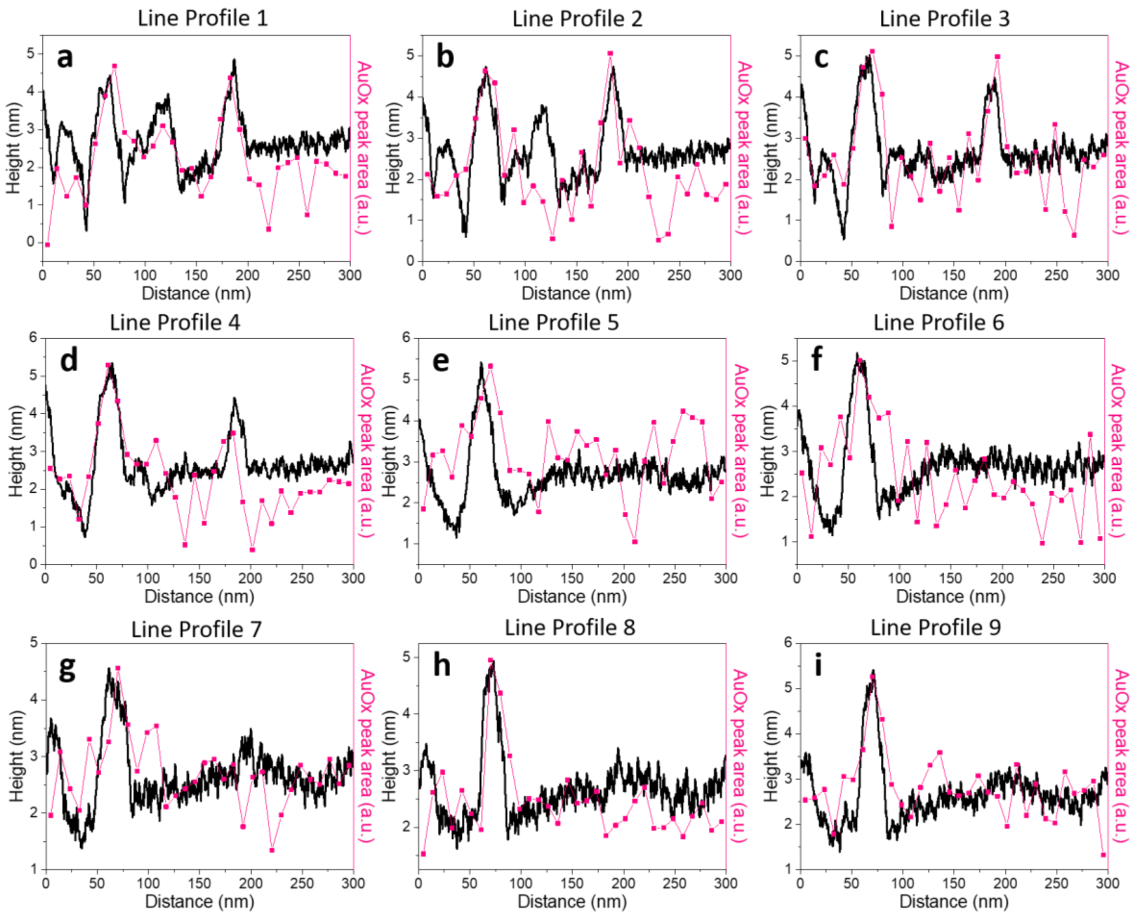

Supplementary Fig. 9 EC-STM and EC-TERS line profiles. Additional line profiles of Fig. 2d correlating EC-STM (black) and EC-TERS AuOx peak area (pink).

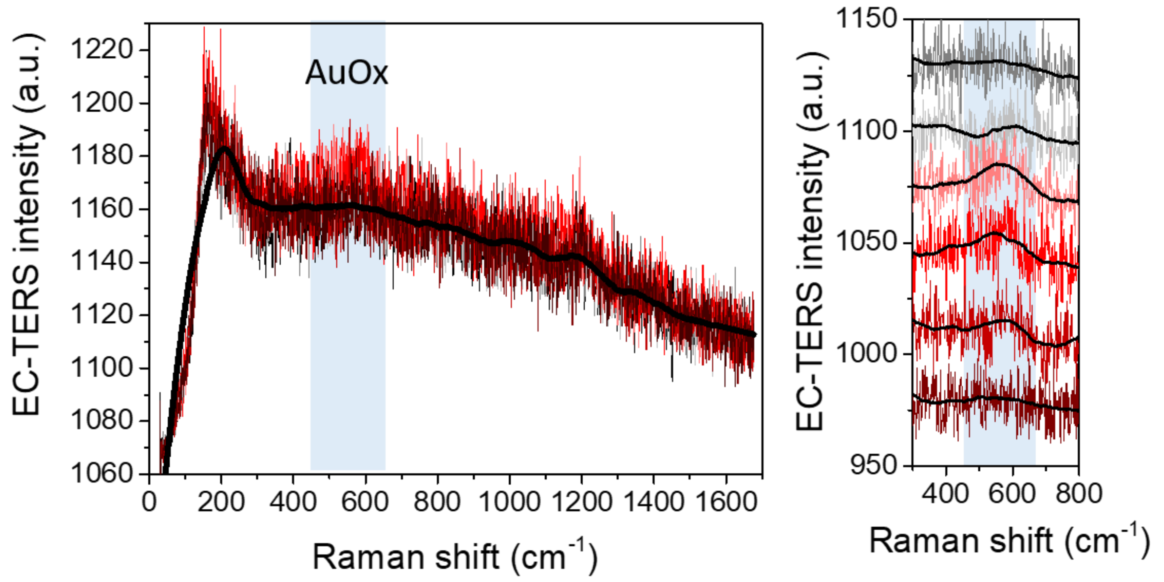

Supplementary Fig. 10 Raw EC-TERS spectra for line profile. Individual spectra from an example TERS line scan starting from a flat Au(111) terrace position (top, black), crossing a selectively oxidized nanodefect (red, middle) and reaching a flat terrace (brown, bottom) on the other side of the nanodefect. The highlighted blue region denotes the gold oxide (AuOx) spectral region.

Supplementary Notes

Supplementary Note 1:

Cyclic voltammetry of Au(111) in sulfuric acid

Supplementary Figure 1 shows a zoom-in image of Fig. 1b in the main manuscript highlighting the different regions characteristic for well-defined Au(111). The yellow region shows the (bi)sulfate adsorption/desorption region, whereas the red region exhibits a clear order/disorder transition peak. The green and blue areas represent the defect oxidation and (111) terrace oxidation regions, respectively. The geometric surface area accounts to $\pi(0.8\ \mathrm{cm}/2)^2 = 0.503\ \mathrm{cm}^2$. Integration of surface charges from 1.225 to 1.6 V vs. Pd-H (full surface oxidation) corresponds to 690.4 µC cm^{-2}, which corresponds well to literature values of around 619 µC cm^{-2} in 1 M H_2SO_4.[1] With an upper integration boundary of 1.45 V vs. Pd-H only 58.7 µC cm^{-2} are detected as only defect structures are oxidized, which is close to ~63 µC cm^{-2} detected by Conway and coworkers[2]. When the fully electro-oxidized Au surface is reduced (integration boundaries 1.6 to 0.85 V vs. Pd-H), the determined charge density of -668.2 µC cm^{-2} is close to the surface oxidation charge value.

Theoretically, one monolayer (ML) AuOx (or ½ ML O_2) amounts to 222 µC cm^{-2} on Au(111) for 1-electron transfer[2]. A complete surface oxidation reaching 6 ML AuOx would amount to 1332 µC cm^{-2}, roughly twice the amount of total charge we measure. Therefore, terrace oxidation likely is limited to the topmost one or two surface (mostly terrace) layers, in agreement with literature[3]. In contrast, the oxidation of the defect sites, however, reaches about 6 ML (see main manuscript text) as inferred from EC-TERS and EC-STM data. The ratio between defect charge density of ca. 60 µC cm^{-2} and total surface charge density thus indicates that the Au(111) single crystal has a defect coverage of ca. 4.5 %.

Supplementary Note 2:

EC-TER spectra for ON and OFF states

The EC-TER spectra from 100 cm^{-1} (dichroic edge filter cut-off at 156 cm^{-1}) to 1700 cm^{-1} corresponding to Fig. 1c in the main text are shown in Supplementary Fig. 2. When defect-catalyzed water splitting is ON, the AuOx stretching mode around 560 to 580 cm^{-1} is detected[4,5].

Small, ill-defined bands can be discerned at ca. 250 cm^{-1} and at ca. 980 cm^{-1} for both ON/OFF states and are tentatively assigned to the 0.1 M sulfuric acid aqueous electrolyte (H_2O and/or SO_4^{2-} modes, respectively). Note that the spectral background does not change when switching between ON and OFF states, indicating that the EC-TERS gap resonance is unaffected by the potential switch.

Supplementary Note 3:

Maintaining the Au-tip oxide free

Within our study, we have taken great care to ensure that the Au-tips stay oxide free and that the obtained gold oxide (AuOx) signal originates from the electro-oxidation at the Au(111) surface. Supplementary Figure 3 shows two cyclic voltammograms (CVs) from the Au(111) electrode (blue) and the Au-tip (black) recorded simultaneously with 0 V bias and a scan rate of 100 mV s^{-1}. As the Au-tip is in principle a defective Au structure, the oxidation starts around the defect oxidation region above 1.3 V vs. Pd-H and exhibits its main peak around 1.45 V vs. Pd-H. On the other hand, the Au(111) shows its strongest anodic peak around 1.55 V vs. Pd-H corresponding to (111) terrace oxidation with a shoulder extending to the defect oxidation region. Importantly, the onset of the reduction of the AuOx at approximately 1.2 V vs. Pd-H clearly overlap for both the Au(111) electrode and the Au-tip. Maintaining the Au-tip potential

at a maximum potential of 1.2 V vs. Pd-H or below thus ensures that the Au-tip resides in the AuOx reduction region and remains AuOx free.

We have achieved this requirement either by applying a constant bias or by keeping the tip potential fixed. For the constant bias, we have utilized a constant bias of 400 mV or higher. For a maximum upper sample potential of 1.6 V vs. Pd-H, this corresponds to 1.2 V vs. Pd-H for the Au-tip (and 400 mV bias). The second approach fixes the Au-tip potential at 1.2 V vs. Pd-H or below, e.g. at 1.0 V vs. Pd-H as for Fig. 2 in the manuscript. It is important to note that fixing the tip potential to a specific potential value inherently changes the voltage bias when switching the working electrode. We have recently investigated that in general a larger bias will lead to an increased tip-sample distance and therefore to a decreased TERS intensity in air, but that this effect is negligible for in-liquid experiments[6]. In any case, when the sample potential is switched from 1.1 to 1.45 V vs. Pd-H, the sample bias voltage increases from 100 to 450 mV, which would (if at all) result in a larger tip-sample distance and a lower EC-TERS enhancement for the ON state compared to the OFF state. Under these conditions, we are able to detect AuOx at around 560 to 580 cm^{-1}, and we do not observe any AuOx intensity at 1.1 V vs. Pd-H.

Supplementary Note 4:

EC-TER spectrum under terrace oxidation conditions

As highlighted in Supplementary Fig. 1, at 1.6 V vs. Pd-H also the (111) terraces are oxidized and not only the defects. Supplementary Fig. 4 shows an example EC-TER spectrum recorded at 1.6 V vs. Pd-H while the Au(111) surface was oxidized at 1.6 V vs. Pd-H. When the potential is changed to 1.15 V vs. Pd-H, the AuOx peak around 560 to 580 cm^{-1} vanishes. Note that conventional Raman (without tip enhancement) cannot resolve the small amount of AuOx surface species formed in the ON state (blue curve).

Supplementary Note 5:

EC-TERS reactivity mapping

The EC-TERS reactivity maps presented in Fig. 2 of the manuscript were obtained in the scan-to-point mode. This scan-to-point mode records the EC-STM image while scanning to the points at which EC-TER spectra are recorded. Due to thermal drift and a spectral acquisition time of 1-3 s, slight shifts in the surface morphology before and after spectral acquisition are apparent. In Supplementary Fig. 5c, this phenomenon is further highlighted. The EC-STM image recorded under reaction conditions at 1.45 V vs. Pd-H exhibits “lines”, at which the EC-TER spectra were acquired. Since the spectral acquisition time is larger than the time required for the EC-STM to record a single line (32 pixels x 1-3 s vs. 1 line s^{-1}), thermal drift is more apparent. For Fig. 2a,c,e and Supplementary Fig. 5a,c, the recorded EC-STM images were flattened according to the standard procedure in Gwyddion (5th polynomial degree). The images were recorded with 1 line s^{-1} scan speed and 512 points per 300 nm distance. The tunneling current setpoint was 1 nA.

The EC-TERS reactivity maps were created as follows: First, the raw EC-TER spectra were Fourier-filtered in matlab using the interactive Fourier Filter (iFilter.m; matlab file exchange) to remove high frequency noise. Supplementary Figure 6a shows example filtered and unfiltered EC-TER spectra. The filter parameters were as follows: frequency = 0.00060677; period = 1648.0761; width = 0.005-0.015; shape = 5. A linear baseline was determined within the boundaries of 440 to 680 cm^{-1} for each individual Fourier-filtered EC-TER spectrum and then subtracted from the Fourier-filtered EC-TER spectrum (Supplementary Fig. 6). Afterwards, the AuOx peak area with the boundaries (440 to 680 cm^{-1}) was determined by simple integration (trapz, matlab). The obtained peak area of the 440 to 680 cm^{-1} peak region was then plotted in OriginPro 9.1 (contour plot). In Fig. 2b, the zero scale bar value was determined by averaging 64 pixels at the OFF state (1.1 V vs. Pd-H) and adding three times the

standard deviation (3649 + 3*656 = 5617). The maximum scale bar value accounts to 17550. In Fig. 2d, the zero scale bar value was set to the average of 64 pixels at a flat Au surface region and adding three times the standard deviation (969 + 3*87 = 1230). The maximum scale bar value accounts to 1715. In Fig. 2f, the zero scale bar value was set to the average of 8 spectra within the flat Au surface region (ON state) and adding three times the standard deviation (5130 + 3*998 = 8124). The maximum scale bar value accounts to 20800. If the zero scale bar value is determined from the OFF state (average from 64 pixels), as for Fig. 2b, the determined zero scale bar value is 6319 (3961 + 3*786). Supplementary Figure 7 compares the resulting maps using both determined zero scale bar values. The EC-TERS maps directly reveal that the three nano-defective structural features in Fig. 2e and Supplementary Fig. 7a,c are most reactive toward the electro-oxidation of gold.

Thermal drift

The thermal drift of our EC-TERS measurements can be estimated from subsequently recorded EC-STM images and the time needed to record the EC-STM images. From the EC-STM images underlying Supplementary Figs. 5a and b, we estimated a thermal drift of ca. 120 nm h^{-1} in x-direction and 45 nm h^{-1} in y-direction. These values correspond well to thermal drift values reported in the literature of about 1-2 nm min^{-1} for EC-STM instruments[7]. EC-TERS intensity profiles in Figs. 3a to c were adjusted to the apparent thermal drift of less than half a pixel.

Supplementary Note 6:

Peak position determination

Supplementary Figure 6b shows an example unconstraint fitting result of the AuOx peak region around 560-580 cm^{-1} with a single Gaussian peak shape using matlab software (peakfit.m, matlab file exchange) after linear background subtraction as described earlier. In this way, the

peak positions of Fig. 4 were determined from the Fourier-filtered EC-TER spectra. The fitting errors were estimated by fitting the unfiltered raw spectra with a bootstrapping analysis incorporated in the matlab software (peakfit.m, matlab file exchange).

EC-TER spectral time-series of surface AuOx

Supplementary Fig. 8 shows an EC-TERS spectral time-series recorded at one position (within the limits of thermal drift, 1-2 nm min^{-1} (see thermal drift section above)) on oxidized Au(111) at 1.6 V vs. Pd-H. The spectral acquisition time was 5 s per spectrum. The acquired EC-TER spectra are reproducible and stable over time, even under electro-oxidation conditions at 1.6 V vs. Pd-H (terrace oxidation).

Additional line profiles and chemical spatial contrast

Supplementary Figure 9 shows additional line profiles across the defective nano-structures of Fig. 2c. EC-TERS is capable to correlate EC-STM electronic topography with EC-TERS chemical information. The defective nano-structures portray reproducibly the largest AuOx EC-TERS signal in comparison to rather flat surface regions. Nano-structures of 20 to 50 nm lateral dimensions can be resolved (Fig. 3a,b and Supplementary Fig. 9). Moreover, Figure 3c demonstrates that for the different step structures the EC-TERS intensity changes sharply within the 9.4 nm pixel size, suggesting that the spatial chemical feature sensitivity is even lower than the pixel size used in this work.

Fundamentals of tip-enhanced Raman spectroscopy (TERS)

TERS is based on the excitation of localised surface plasmons (LSPs) in the apex of a metal (or metallized) SPM tip. In our configuration, we employ a commercially available electrochemical scanning tunnelling microscope (EC-STM) coupled to a Raman optical platform as described

in the Methods section in the main text. The working principle of TERS is based on the efficient coupling of a focused laser beam with the tip to excite LSPs, which generates a strong electromagnetic near-field at the very apex of the STM tip. In a way, the tip acts as an antenna and converts, or “concentrates”, far-field radiation into a nm-confined field underneath the tip. The near-field character of the Raman excitation provides the extreme spatial optical resolution, typically of a few nm for in-air experiments, which depends essentially on the size, curvature and surface (atomic) topography of the tip apex.

The created near-field typically is a factor 10 to 100 larger than the excitation far-field and can induce Raman scattering in the species or molecules located at nm-close distance below the STM tip. The Raman scattering intensity, in a first approximation, scales with the fourth power of the magnitude of the excitation field. The strong field enhancement at the tip compared to the far-field intensity enables the detection of very few surface scatterers down to single molecules. When the substrate is a metal or exhibits metal optical properties similar to the ones of the tip, LSP excitation in the tip is accompanied by a corresponding image dipole formation in the substrate which leads to even higher field enhancements in the tip-sample gap. Accordingly, such a TERS configuration is called gap-mode TERS and provides typically even higher sensitivity than TERS on a dielectric substrate.

The interested reader is referred to recent reviews about TERS, its fundamentals and applications[8,9].

TERS intensities and plasmonic gap properties

In general, Raman intensities are governed by the local field strength – for TERS, this corresponds to the local field enhancement, or near-field strength, in the tip-sample gap – and by the amount of scatterers for a given experimental configuration, detection sensitivity and

scattering cross section of the system under study. The effect of the formation of a plasmonic tip-sample gap on the field (enhancement) can be seen (albeit not quantified as there is no signal in the conventional Raman spectrum) from the comparison of the conventional (no tip-sample gap) and TERS signals as shown in Supplementary Fig. 4.

For self-assembled monolayers of organic molecules adsorbed at metal substrates, sometimes a local increase in TERS intensity between a factor five to ten at step edges or surface protrusions compared to TERS at neighbouring flat Au regions, partly also accompanied by frequency shifts of Raman modes, has been reported in the literature[10-15]. Such edge effects can be attributed to local heterogeneities in the plasmonic properties of the formed tip-sample gap that differ depending on the actual (atomic) gap geometry and field localisation and polarisation. As a result, the LSP or gap resonance shows nanoscale spatial heterogeneities both in intensity and in location of the resonance maximum causing differences in coupling efficiency between excitation far-field laser and gap mode. Strongest near-fields are created under resonance conditions when the excitation wavelength approximately matches the LSP resonance maximum. Therefore, shifts in the gap plasmonic resonance maximum (intensity and position) can lead to significant differences in TER scattering intensities. Furthermore, highly localised strong fields can lead to a local Stark effect that causes shifts in vibrational frequencies[12]. Also different adsorption geometries of molecules at step edge sites compared to terrace sites can account for differences in TER shifts[13].

In general, the plasmonic (gap) properties can be deduced from the shape (plasmon resonance energy) and the intensity (field strength, or enhancement) of the spectral background[16].

Supplementary Figure 2 shows a comparison between TER signals recorded in ON and OFF conditions. Evidently, the background does not differ, neither in shape nor in intensity. As discussed in the main text of the manuscript, changes in the tip-sample distances and thus in the field enhancement upon variation of the tip-sample bias (due to the variation in applied

potential), have previously been shown to be negligible in water[26], in line with the conclusion drawn from Supplementary Fig. 2 about the unaltered field enhancement in air compared to in water. As such, it is fair to assume that the field strength and enhancement do not change upon potential change and that the obvious change in peak intensity is due to a change in the amount of scatterers present in the nearfield, i.e. the amount of AuOx formed.

Furthermore, Supplementary Figure 10 shows raw data from an example line scan. From these spectra it is evident that the TERS background recorded at oxide-free terrace sites and the background recorded on the oxidized defect are identical, i.e there is no measurable difference between the plasmon resonance energy or field enhancement above terrace or defect sites. As such, we have attributed the oxide intensities recorded on defect sites and their spatial variation across defects to a variation in the local number of AuOx scatterers, or the thickness of the formed defect oxide layer.

Regarding the spatial uniformity of the background signal, we also refer to Supplementary Fig. 5e. Here, the EC-TERS map without background subtraction (field enhancement and number of scatterers determine intensity) is plotted – and is essentially the same as the one presented in the main text in Fig. 2d after background correction to present a "pure" chemical map corrected for (however small) near-field spatial variations (number of scatterers determine change in peak intensity). Comparing Supplementary Fig. 6e and Supplementary Fig. 2d, it is evident that, independent of whether with or without background correction, the quantitative result of selective AuOx formation on the defects remains the same.

In summary, while edge effects due to spatially heterogeneous plasmonic gap properties were observed in TERS imaging of self-assembled monolayers, our data does not provide any indication for measurable differences in tip-sample coupling neither as a function of tip position nor of applied potential within the employed potential window. Therefore, we attribute the TER intensity differences to spatial- and/or potential-dependent variations in the amount of AuOx

scatterers.

Supplementary References

1. Zhumaev, U. *et al.* Electro-oxidation of Au(1 1 1) in contact with aqueous electrolytes: New insight from in situ vibration spectroscopy. *Electrochim. Acta* **112**, 853–863 (2013).

2. Angerstein-Kozlowska, H., Conway, B. E., Hamelin, A. & Stoicoviciu, L. Elementary steps of electrochemical oxidation of single-crystal planes of Au-I. Chemical basis of processes involving geometry of anions and the electrode surfaces. *Electrochim. Acta* **31**, 1051–1061 (1986).

3. Peuckert, M., Coenen, F. P. & Bonzel, H. P. On the surface oxidation of a gold electrode in 1N H_2SO_4 electrolyte. *Surf. Sci.* **141**, 515–532 (1984).

4. Desilvestro, J. & Weaver, M. J. Surface structural changes during oxidation of gold electrodes in aqueous media as detected using surface-enhanced Raman spectroscopy. *J. Electroanal. Chem.* **209**, 377–386 (1986).

5. Shi, H., Asahi, R. & Stampfl, C. Properties of the gold oxides Au_2O_3 and Au_2O: First-principles investigation. *Phys. Rev. B* **75**, 1–8 (2007).

6. Martín Sabanés, N., Elizabeth, A., Pfisterer, J. H. K. & Domke, K. F. The effect of STM parameters on tip-enhanced Raman spectra. *Faraday Discuss.* **205**, 233–243 (2017).

7. Chen, C., Hayazawa, N. & Kawata, S. A 1.7 nm resolution chemical analysis of carbon nanotubes by tip-enhanced Raman imaging in the ambient. *Nat. Commun.* **5**, 3312 (2014).

8. Shi, X., Coca-López, N., Janik, J. & Hartschuh, A. Advances in tip-enhanced near-field Raman microscopy using nanoantennas. *Chem. Rev.* **117**, 4945-4960 (2017).

9. Pozzi, E.A., Goubert, G., Chiang, N., Jiang, N., Chapman, C.T., McAnally, M.O., Henry, A.-I., Seideman, T., Schatz, G.C., Hersam, M.C. & Van Duyne, R.P.

Ultrahigh-vacuum tip-enhanced Raman spectroscopy. *Chem. Rev.* 117, 4961−4982 (2017).

10. Zhang, W., Cui, X., Yeo, B.-S., Schmid, T., Hafner, C. & Zenobi, R. Nanoscale roughness on metal surfaces can increase tip-enhanced Raman scattering by an order of magnitude. *Nano Lett.* **7**, 1401-1405 (2007).

11. Chen, J., Yang, W., Dick, K., Deppert, K., Xu, H. Q., Samuelson, L. & Xu, H. Tip-enhanced Raman scattering of *p*-thiocresol molecules on individual gold nanoparticles. *Appl. Phys. Lett.* **92**, 093110 (2008).

12. Marr, J.M. & Schultz, Z.D. Imaging electric fields in SERS and TERS using the vibrational Stark effect. *J. Phys. Chem. Lett.* **4**, 3268-3272 (2013).

13. Bhattarai, A., Joly, A.G., Hess, W.P. & El-Khoury, P. Visualizing electric fields at Au(111) step edges via tip-enhanced Raman scattering. *Nano Lett.* **17**, 7131-7137 (2017).

14. Wang, R. & Korouski, D. Elucidation of tip-broadening effect in tip-enhanced Raman spectroscopy (TERS): A cause of artefacts or potential for 3D TERS. *J. Phys. Chem. C* **122**, 24334-24340 (2018).

15. E. Sheremet, E., Ki, L., Stepanichsheva, D., Kolchuzhin, V., Milekhin, A., Zahn, D.R.T. & Rodriguez, R.D. Localized surface curvature artefacts in tip-enhanced nanospectroscopy imaging. *Ultramicroscopy* **206**, 112811 (2019).

16. Lin, K.-Q., Yi, J., Zhong, J.-H., Hu, S., Liu, B.-J., Liu, J.-Y., Zong, C., Lei, Z.-C., Wang, X., Aizpurua, J., Esteban, R. & Ren, B. Plasmonic photoluminescence for recovering native chemical information from surface-enhanced Raman scattering. *Nat. Commun.* **7**, 14891 (2017).
